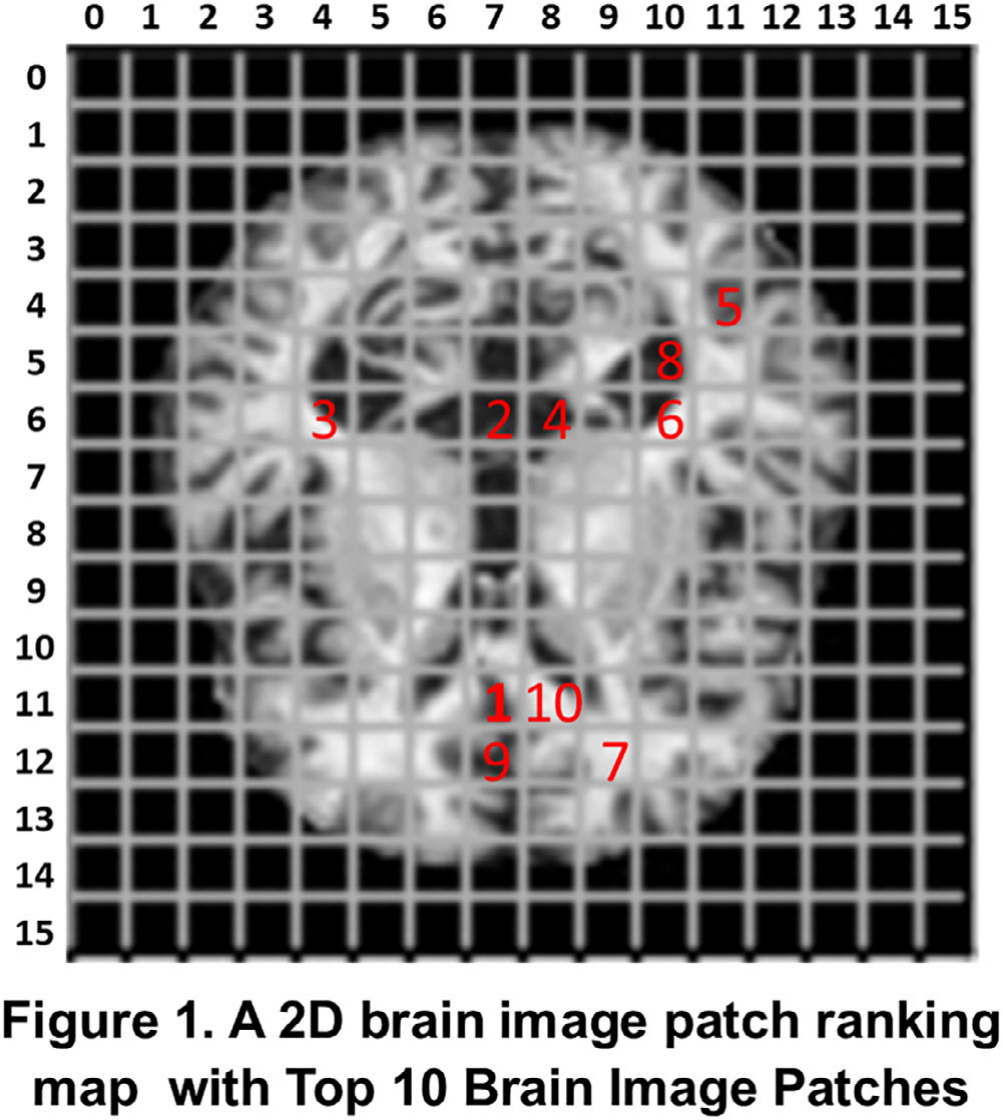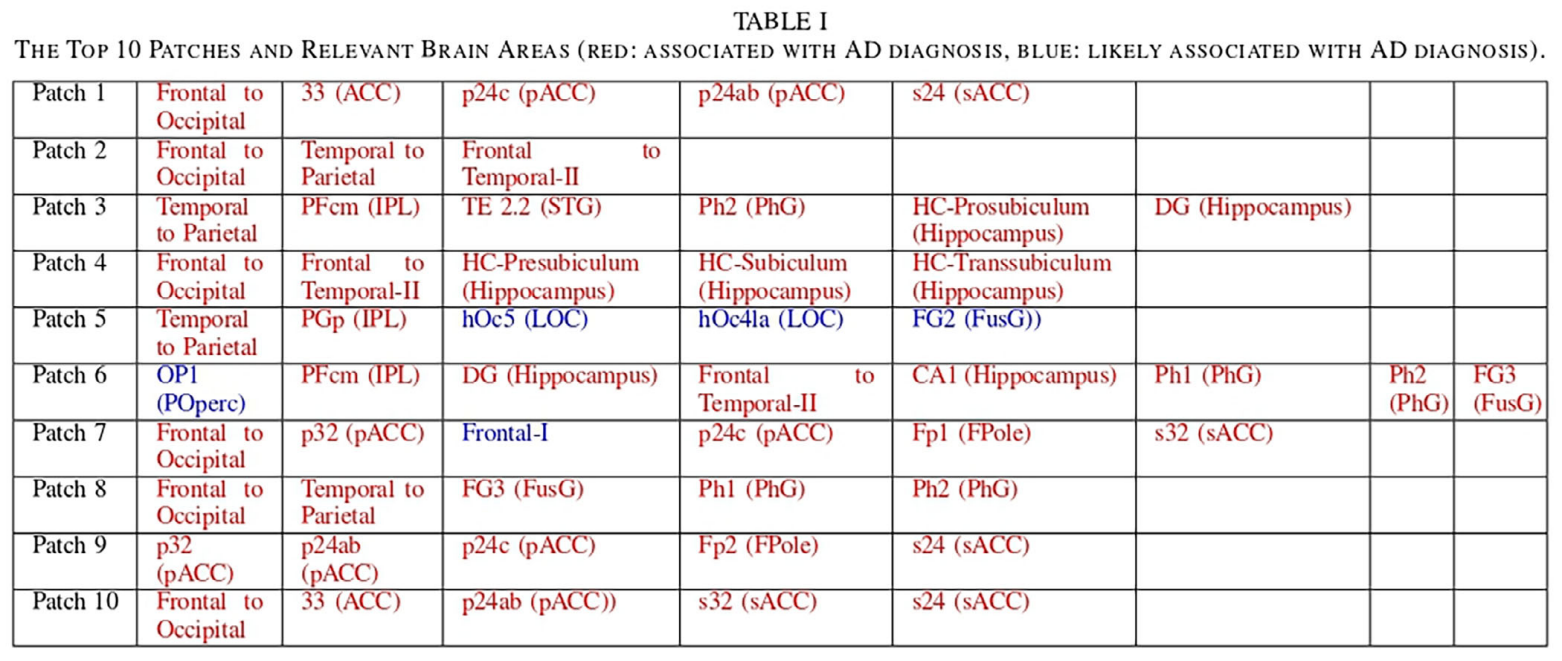# The robust 2D brain image patch ranking algorithm with feature selection, heatmaps and ChatGPT for interpretable Alzheimer's disease diagnosis

**DOI:** 10.1002/alz70861_108992

**Published:** 2025-12-23

**Authors:** Luna Mingyi Zhang

**Affiliations:** ^1^ Stony Brook University, Lake Grove, NY USA

## Abstract

**Background:**

An important problem is discovering and understanding the reasons behind Alzheimer's disease (AD) diagnosis from analyzing brain images. We can use computer vision techniques to find the relationship among top‐ranked 2D brain image patches, relevant feature sets, associated brain areas, and the disease diagnosis. A new robust 2D image patch ranking algorithm is created to generate a reliable 2D patch ranking map (PRM) displaying top‐ranked 2D brain image patches for explainable AD diagnosis.

**Method:**

An efficient convolutional neural network (CNN) model consisting of convolutional layers, a new feature selection (FS) layer, and a classifier is built. The new FS algorithm is made to reliably select the top common features from different top feature sets. The new FS‐Grad‐CAM method is developed to generate explainable heatmaps with a smaller number of highlighted areas associated with top‐ranked features. Two new feature matrices and two new heatmap matrices are used to reliably rank patches to better explain the relationship among patches, top‐ranked features, relevant top‐ranked feature maps, and the image classifications.

**Result:**

The AD MRI preprocessed dataset for 4‐class image classification with 6,400 128x28 axial brain images is used for simulations. The associations between brain areas and AD are analyzed by using hybrid information from both relevant publications and ChatGPT. Simulation results show that the top 10 patches (i.e., 3.9% of all 256 patches) are associated with 40.4% of all 57 brain areas associated with AD, 11.4% of all 44 brain areas likely associated with AD, and 0% of all three brain areas not likely associated with AD, as shown in Table 1. The efficient CNN with FS can have higher classification accuracy, smaller model size, and higher explainability than the conventional CNN. Figure 1 shows a PRM with top 10 brain image patches associated with relevant brain areas for AD diagnosis.

**Conclusion:**

The new robust 2D image patch ranking algorithm can reliably generate the 2D PRM for interpretable AD diagnosis. A medical doctor may conveniently use the 2D PRM to understand the relationship among top‐ranked 2D image patches, relevant feature sets, important associated brain areas, and AD diagnosis.